# Productivity and stress recollection inaccuracy: Anchoring effects in work-from-home evaluation

**DOI:** 10.1371/journal.pone.0320959

**Published:** 2025-04-03

**Authors:** Martijn Stroom

**Affiliations:** Department of Finance, School of Business and Economics, Maastricht University, Maastricht, The Netherlands; Fondazione Policlinico Universitario Agostino Gemelli IRCCS, Universita' Cattolica del Sacro Cuore, ITALY

## Abstract

Self-reported productivity and satisfaction have become central metrics in evaluating work-from-home (WFH) policies, yet their reliability remains largely unexamined. Despite growing scrutiny of WFH efficacy, assessments continue to rely heavily on subjective evaluations, creating a persistent gap between perceived and objective productivity measures within the working-from-home literature. This study investigates whether retrospective self-reports of productivity during WFH are systematically biased due to recollection inaccuracy, particularly through anchoring biases in memory recall. A two-wave survey data sample consisting of 772 home-workers during the 2020 shift to the home office examines recollection accuracy as well as the underlying mechanism. Using a five-factor productivity scale, within-subject analyses explore consistency within and between multiple waves and evaluate the predictive value of the targeted score compared to the current (inaccurate) score. The signed rank test shows that recollection scores consistently underestimate past scores (one factor: p < .002; four factors: p < 0.0004). The recollected scores are closer to the current score than the targeted score (for all factors: p < .0004) and have a greater magnitude impact on the recollection scores than the targeted score (difference in OLS coefficients ranging from.164 to.606). Exploration of trends additionally suggests that, although the absolute scores are influenced by the current reference point, the relative changes seem consistent over time. These findings highlight the risks of relying on self-reported retrospective productivity measures in shaping WFH policies. The observed biases help contextualize the ongoing discrepancy between optimistic self-reports and more pessimistic objective measures of WFH productivity. Without accounting for recollection biases, assessments of WFH effectiveness may be flawed, potentially leading to suboptimal or counterproductive policy decisions.

## Introduction

The COVID-19 pandemic incited a seismic shift in organizational structures globally, thrusting traditional on-site work models towards remote work paradigms [1–5]. This swift and massive migration to remote work environments ushered in a new era of labor policies and practices, in which employers grappled with novel challenges without the luxury of pilot testing. A renewed emphasis was placed on understanding employee well-being [6], job satisfaction [7], and retention [8] during this turbulent transition, with self-reported productivity and satisfaction taking center stage as the principal barometers of work-from-home (WFH) efficacy [2,6,9–11]

Initial reports based on self-reported productivity found a positive impact of productivity of the WFH shift [9–11]. Barrero et al. [12] recently underlined that workers perceive the working-from-home shift to positively impact their productivity with 7.4%, based on the response of 13,082 US employees to “How much less/more efficient are you working from home than on business premises?”. Later publications measuring changes in objective productivity find, however, that WFH does not consistently lead to improved productivity and often shows negative effects [13–15]. Gibbs et al. [14] even found that workers who preferred WFH were 12% faster and more accurate at baseline (before the shift to WFH) but experienced a 27% decrease in productivity when working from home compared to their office performance, leading to a net productivity drop of 15%. In contrast, those who preferred office work experienced a smaller net productivity decline of 13% when working from home. The authors argue that self-reported productivity may be biased if employees enjoy and hope to retain WFH. In reality, however, this preference could be correlated with decreased productivity, as Emanuel & Harrington [15] find a negative self-selection into WFH. Together, this points to a disconnect between studies highlighting self-reported preferences and productivity for WFH and those analyzing objective productivity outcomes, revealing a significant knowledge gap.

The discrepancy between stated preferences, self-reported productivity, and objective measures has not gone unnoticed in the academic debate. Barrero et al. [12] attribute this discrepancy to confusion between full-time and part-time WFH scenarios but fail to support that empirically as their survey does not specify or imply part-time WFH itself, a prerequisite for a positive effect on productivity according to their own reasoning. Meanwhile, prior claims of productivity gains from remote work are being moderated, with more recent findings indicating that even a partial shift to WFH has no significant impact on productivity [16]. This ongoing reassessment highlights the variability in conclusions drawn from predominantly self-reported productivity over time. Since employers are estimated to save an average of $11,000 each year per half-time remote worker, clarification on the reliability of subjective self-reported WFH productivity is paramount in order to create a sustainable and profitable WFH policy [17]. Thus, this paper sets out to investigate the accuracy and relevance of self-reported productivity and related factors over time during a prolonged work-from-home period.

Accurately self-reporting productivity requires individuals to meticulously recount their own past experiences. Many strands of literature have previously questioned the accuracy of autobiographical memory and highlighted the influential role of memory biases [18–21]. Recent research has distinguished between two key dimensions of recollection failure: the success of retrieval and the precision of recollection [22]. Retrieval failures occur when memories fail to reach the threshold for being consciously recalled, whereas inaccuracies in precision arise from errors in reconstructing details of the memory [23,24]. Although WFH is likely to meet the recollection threshold due to its salience, the precision of the recollection is not guaranteed. Periods of significant disruption, such as the COVID-19-driven shift to WFH, are likely to destabilize memory retrieval and reconstruction [25–28]. The novelty of the COVID-19 or WFH experience could further induce recollection inaccuracy, as recollection processes are generally heavily interrelated with familiarity [29,30].

While contextual factors such as salience, disruption, or familiarity may shape the recollection of WFH experiences, structural biases can distort the self-reported accuracy. The influence of contextual factors on WFH recollection could diminish over time, yet systematic errors in memory recall, shaped by consistent internal or external influences rather than random or purely contextual factors [31], might persist. These biases become particularly pronounced in emotionally charged situations, in which individuals’ mood and affect can strongly influence how they remember past experiences [32,33]. For example, emotional regulation could explicitly reshape memories to ensure their alignment with their emotional well-being, self-image, or coping strategies, allowing individuals to construct narratives that support their current psychological and emotional needs [19,34]. More independent of individuals’ differences, current states may color the perception of past states, leading to a tendency for the present state of mind to carry over into recollections of past experiences [35,36]. These findings highlight that recollection-based productivity might not only be influenced by temporary contextual factors, such as novelty, but could also be systematically distorted by the preservation of one’s self-image or one’s current state.

How and in which direction these potential biases would skew the self-reported WFH productivity and satisfaction remains unclear a priori. Temporal self-appraisal theory has suggested that individuals tend to evaluate past achievements as inferior to their present capabilities, exaggerating perceived personal growth over time [37–39]. Ross [40] assessed multiple strains of research and suggested that the magnitude of differences between past and present states was at times exaggerated to emphasize change or underestimated to emphasize consistency over time. Dual recollection theories, proposing that accurate recollection depends on both reconstructing the target memory as well as its context at that time [41], further complicate the prediction of the intensity of recollection inaccuracies during WFH.

One known bias that could help identify these inaccuracies, their structural nature, and help predict the impact is anchoring. Anchoring in recollection occurs when individuals rely disproportionately on their current state as a reference point for recalling past experiences [42,43]. This is consistent with the structural tendency of an individual’s present state-of-mind to color and distort their memories [35,36]. Consequently, recollected values are systematically biased towards the present values. For example, current productivity levels may serve as potent cognitive anchors that carry over into recollections of past performance. Unlike random errors, anchoring biases are structural and predictable [20,21], creating both challenges for interpreting self-reports and opportunities for identifying the underlying causes of inaccuracies in WFH productivity assessments.

Together, the existence of similar and structural recollection inaccuracy might amplify the perceived shift in productivity associated with the transition to WFH. Unfortunately, these insights seem overlooked in productivity literature, a neglect that may have far-reaching implications in the current scenario where the reliance on self-reported productivity is steadily increasing. In response to these concerns, this paper investigates the accuracy of self-reported productivity and related factors over a prolonged WFH period. Specifically, it examines whether individuals’ recollections of past productivity are more influenced by their current productivity rather than reflecting the actual past productivity. This *anchoring* bias is explored across various WFH factors, such as productivity, stress, and non-work satisfaction. The paper further investigates whether this inaccuracy is structural, thus affecting the *absolute* levels of recollected scores while still preserving *relative* trends over time, or whether it represents random error, rendering recollections unreliable for assessing both levels and trends.

The findings in this paper reveal that participants’ recollections of their earlier WFH experiences are systematically biased. After establishing that participants report significant changes in WFH-related factors over time and between two measurement moments (H1 and H2, respectively), the paper shows that recollected scores are more strongly influenced by the current scores than by the target scores (H3), suggesting an anchoring bias. Regression analyses confirm this bias, showing that recollections align more closely with participants’ present conditions than with their past experiences. Despite these inaccuracies in absolute levels, further analysis reveals that recollected trends over time remain stable, with the deltas between multiple recollected points showing consistent patterns across measurement periods. This suggests that while recollection is unreliable for assessing specific levels, evaluating changes over time remains informative. These findings highlight critical considerations for the use of recollection-based self-reports in assessing WFH outcomes.

## Methods

### Sample

Dutch workers with jobs suitable to perform at home between 18 and 67 years old who worked at least part-time from home at the time of the survey were approached via Flycatcher. Flycatcher is ISO 20252 qualified and manages an academic research-oriented, high-quality panel of over 10,000 active participants closely representing the cross-section of the Dutch population on relevant demographics. Only Dutch workers are included since the COVID-19 working-from-home policies were at risk of rapid change between waves between countries, yet policies within the Netherlands were similar for all Dutch workers during the data collection period. Panel members without work, previously without work, or working exclusively from the office are excluded from the sample.

**Fig 1:**
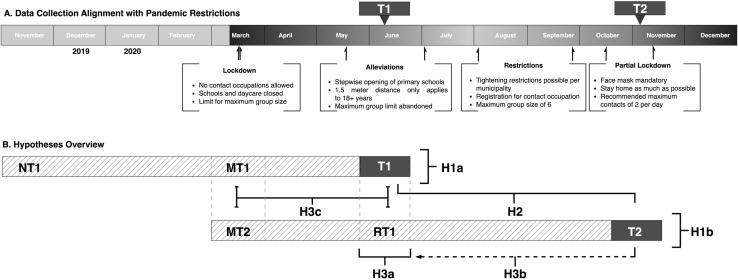
Measurement waves and schematic overview hypotheses. Fig. 1a illustrates the measurement periods (T1 and T2) in relation to the intensity of pandemic restrictions in the Netherlands during each respective time. Fig. 1b expands on this by showing the measurement periods (T1 and T2) along with the retrospective periods for each (T1: MT1 and NT1; T2: RT1 and MT2, respectively). Additionally, it schematically represents the relationship between each hypothesis and the targeted scores collected at T1 or T2. Both panels (Fig 1a and Fig 1b) are scaled proportionally to one another.

Data was collected during two waves: the first from June 24 to July 1, 2020, and the second from November 30 to December 10, 2020. Fig 1a shows that these measurement periods were selected as they correspond to relatively comparable phases of pandemic restrictions. The June 2020 measurement occurred at the start of the gradual alleviation of stricter restrictions, while the November 2020 measurement coincided with the onset of tighter restrictions. Without external control, these periods presented the most reasonable similar conditions in work-from-home policies and social distancing practices (e.g., work-from-home was the national policy, there were no common holidays during these periods, and restrictions implied similar social consequences). The rapid timing of the two waves ensured that working from home remained a novel and dynamic experience. This approach increased the likelihood of capturing meaningful within-person differences in perceived productivity and work happiness that could drive variations in WFH success, enabling the investigation of recollection accuracy, while minimizing the risk of structural adjustments that might introduce endogeneity into these variables. Hypothesis 1 (a and b) and 2 explore whether meaningful within-person differences in WFH experiences indeed exist.

The first wave reached 1,048 participants, and the second wave reached 1,002 participants via the Dutch Flycatcher panel. In total, 772 participants out of 1,278 unique participants completed both waves and are included in this paper (attrition rate of 26%). This sample, described in S1 Table, consisted of workers ranging in age from 20 to 68 (M = 44.27, SD = 12.46), of which 58% were male. The average education level is noticeably higher in the sample, where 54% have obtained at least an undergraduate degree, than in the Dutch population, where only 35% have obtained at least an undergraduate degree. This can be attributed to the fact that the degree to which work can be executed from home is likely correlated with more cognitively skilled occupations. Participation was reimbursed. All members of the Flycatcher panel provide written informed consent, are allowed to drop out at any time, and included participants have actively consented to participation (‘double-active-opt-in’). The research setup was reviewed and approved by Maastricht University's Ethical Review Committee Inner City Faculties (ERCIC_195_09_06_2020).

### Instrument

Five dimensions of productivity were assessed using the self-report-based WFH-HWQ questionnaire [44]. This questionnaire is a reconstructed and validated variation of the Health and Work Questionnaire specifically fitting the work-from-home [45]. The included factors are Productivity, Productivity by Others, Peer Relations, Nonwork Satisfaction, and Stress and Irritability. The WFH-HWQ factors explicitly include the presenteeism orientation of productivity. However, Stroom [44] argues that the factor Stress and Irritability additionally approximates absenteeism due to its correlation with both burnout and absence from work indicators.

### Data collection

In order to monitor the productivity and work satisfaction during the transition from the office to work, participants are asked during two waves to report on productivity and stress factors at that time (June 2020: **T1**, and November 2020: **T2**) as well as recollect the scores on the factors for two previous periods.

Fig 1b shows the two measurement points, T1 and T2, and their recollected scoring periods. For instance, participants scored all questions for their current state during June 2020 (**T1**), for the beginning of the pandemic in March (**M**arch scored at **T1**: **MT1**), and before the pandemic (**N**ovember 2019 period **T1**: **NT1**). Similarly, participants were asked to score their current state during November 2020 (**T2**), during June 2020 (**R**ecollection of **T1**: **RT1**), and during the beginning of the pandemic in March (**M**arch period score at **T2**: **MT2**). This structure enables a comparison between a direct measure (T1) with a retrospective measure (RT1) and two retrospective measures for the same period (MT1 versus MT2).

Note that additional questions were included in the survey for a separate study on the effect of the indoor environment on productivity [46]. It is reasonable to assume that the indoor environment satisfaction might influence (perceived) productivity, yet it is unlikely to influence recollection, thus not interfering with the results of this paper. Similarly, the pre-pandemic (NT1) score enabled a comparison between work and home office, and will thus not be discussed further in this paper due to the lack of comparative values.

### Hypothesis and analysis

This paper describes the self-reported trend over time for each separate measurement, the within-sample development between the two current measurements (T1 and T2), and investigates the recollection accuracy. Fig 1b schematically shows an overview of these three main hypotheses.

The first two hypotheses explore whether work-from-home affects perceived productivity. The pandemic regulations spurring WFH facilitated an opportunity to collect meaningful within-person differences in perceived WFH success, which in turn enables the unique investigation of workers’ recollection accuracy. Hypothesis 1 tests whether participants indeed report significant changes over time in WFH-related factors over multiple time points. This is tested twice (H1a and H1b), once for each measurement period (T1 and T2, respectively). Specifically, hypotheses 1a and 1b are examined using a Friedman test, a non-parametric repeated-measure within-subject ANOVA alternative. Significant differences are further examined in a post-hoc analysis using the extended Mantel-Haenszel (Cochran-Mantel-Haenszel) stratified test of association. Note that most WFH factors of interest in this study are bounded between 0 and 10, with averages typically ranging between 6 and 8. Due to the large sample size, standard statistical tests for normality often yielded significant results, despite kurtosis tests frequently being non-significant, though not consistently. However, visual inspections, such as Q-Q plots, suggested that the data approximated normality. To adopt a conservative approach, non-parametric tests were applied given their robustness for bounded and slightly skewed data. For consistency, test-related statistics are reported as medians and interquartile ranges (IQR).

Hypothesis 2 determines whether the current scores of all factors changes between the two measurement periods (T1 ≠  T2), using a within-subject non-parametric Wilcoxon signed rank test. While H1 focuses on variation within recollected scores for the same measurement period, H2 tests a repeated-measures perspective by comparing current scores between T1 (June 2020) and T2 (November 2020). In addition to underlining the relevance of recollection reliability, identifying differences between the target scores (T1) and the current scores (T2) may enhance the ability to disentangle the relative influence of each score on recollection, as even the presence of small differences allows for exploration of these relationships.

While H1 focuses on variation within recollected scores for the same measurement period, H2 tests a classic repeated-measure consistency perspective by comparing current scores between two distinct measurement periods. Establishing variation between the target scores (T1) and the current scores (T2) is critical for identifying the primary source of recollection inaccuracy between these two points. Without differences between T1 and T2, it would be impossible to meaningfully disentangle which score influences recollection.

Hypothesis 3 focuses on the main question of the paper: do we observe recollection inaccuracy, and if so, what are the mechanisms behind these recollection inaccuracies? H3a first tests whether recollected scores (RT1) are significantly inaccurate from the targeted scores (T1). H3b continues by testing whether this inaccuracy results from recollected scores (RT1) being systematically biased toward the current scores (T2) rather than reflecting the target scores (T1), in line with the anchoring bias [31,34,42,43]. Hypothesis 3c addresses whether recollection inaccuracies follow a consistent pattern over time by including two recollected scores (MT1 and MT2, respectively collected during T1 and T2). Structural recollection inaccuracies would imply that all recollections are biased in a similar (stable) way, rather than introducing random noise. According to Schacter [20,21], anchoring biases differentiate from random errors due to their structural and predictable nature. The consequence of confirming that the identified bias is structural is that the reported recollected trends remain informative. Note that hypothesis 3b identifies a recollection bias as it compares a recollection score with a current score measured at that time, whereas H3c compares two recollection measures, MT1 and MT2, without an objective ‘current’ score. H3c thus tests the continuation of over- or underestimation across time without anchoring it to a specific point of reference.

Hypothesis 3 is examined in three steps. First, a within-subject non-parametric Wilcoxon signed rank test whether RT1 significantly differs from T1 (H3a). Accurate recollection would be reflected by an insignificant difference between the recollection of T1 in the T2 measure (**R**ecollection **T1**: **RT1**) and the actual scores at that time (T1).

Second, the underlying mechanism of the recollection inaccuracy is explored by testing hypothesis 3b. Specifically, it tests whether the recollection of RT1 is more often closer to the targeted score (past score: T1) or the carried over score (current score: T2). Using a binomial test, the absolute delta of RT1 and T1 is compared with the absolute delta of RT1 and T2 for each individual *i* and WFH-HWQ factor *j*. The absolute delta shows the net inaccuracy: the higher the absolute delta coefficient, the higher the deviation between T1 and RT1 scores. If the absolute error between RT1 and T1 is larger than the absolute error between RT1 and T2, RT1 is biased towards the wrong, current score T2. Note that this indicator excludes observations for which T = T2 = RT1, as the lack of variation prohibits the identification of the distinction between accuracy and inaccuracy. This condition excluded 9% of the observations on average per factor and 4% for Productivity specifically.

A dummy variable indicates if the absolute error between RT1 and T1 is smaller than the absolute error between RT1 and T2 (eq. 1).


$$\begin{array}{l} {\text{Indicator }}_{ij}=1\text{ if }\left|\Delta {\left(RT1-T1\right)}_{ij}\right|<\left|\Delta {\left(RT1-T2\right)}_{ij}\right|\\ {\text{Indicator }}_{ij}=0\text{ if }\left|\Delta {\left(RT1-T1\right)}_{ij}\right|>\left|\Delta {\left(RT1-T2\right)}_{ij}\right| \end{array}$$ (1)

The binomial test assumes the inaccuracy ratio between RT1 and either T1 or T2, indicated by the indicator variable, to be random (.50). This assumption is conservative, as RT1 is explicitly aimed at recalling T1. Since a deviation towards T1 is to be expected, a significant deviation towards T2 would thus underestimate the strength of the recollection inaccuracy.

Hypothesis 3b is further investigated in order to compare the predictive strength and control for individual heterogeneity. Multiple linear regressions (MLR) estimate the predictive strength of both the target score (T1) and the current score (T2) on the retrospective score (RT1) for all respective WFH-HWQ factors using the following model:


$$RT1_{j}=\alpha_{0}+\alpha_{1}T1_{j}+\alpha_{2}T2_{j}+\alpha_{3}Controls_{j}+\varepsilon_{j}$$ (2)

For each WFH-HWQ factor, this model estimates to what extent each respective WFH-HWQ factor *j* is predicted by the score of that same WFH-HWQ factor at T1 and T2.

Hypothesis 3c further investigates whether this bias is structural by examining whether relative trends across two recollected periods remain consistent, despite inaccuracies in absolute levels. Specifically, this hypothesis tests whether recollection errors affect all recollected scores in a similar way, indicating that the anchoring bias operates consistently and structurally across time. If the trends show consistency, it suggests that recollections remain informative for identifying relative changes, even if the absolute levels are biased. First, a pairwise Wilcoxon signed rank test explores whether the inaccuracy between T1 and RT1 is related to the difference between MT1 and MT2 within-subject (eq. 3). This analysis explores whether participants’ relative scores are unaffected (or absolute scores are similarly biased) by current scores.


$$\Delta {\left(RT1-T1\right)}_{ij}\neq \Delta {\left(MT2-MT1\right)}_{ij}$$ (3)

Similarly to the approach of H3b, a linear regression estimates whether the deltas between the scores for June (T1 and RT1) are related to the deltas between the scores for March (MT1 and MT2) and compares the predictive strength whilst allowing for individual controls. Model 4 estimates the predictive value of the absolute delta between the current scores on the absolute delta between both retrospective scores:


$$\left|\Delta {\left(\text{M}T1-MT2\right)}_{i}\right|=\beta_{0}+\beta_{1}\left|\Delta {\left(T1-RT1\right)}_{i}\right|+\beta_{2}Controls_{i}+\varepsilon_{i}$$ (4)

For all non-parametric paired comparison tests, I apply the most conservative multiple testing procedure since I consistently test 5 different factors (e.g., Bonferroni correction) [47]. All paired tests in this paper are subject to the following threshold conversion: α=5% equals 0.01 (*), α=1% equals 0.002 (**), and α=0.1% equals 0.0004 (***).

## Results

### Descriptive trends over time

**Fig 2:**
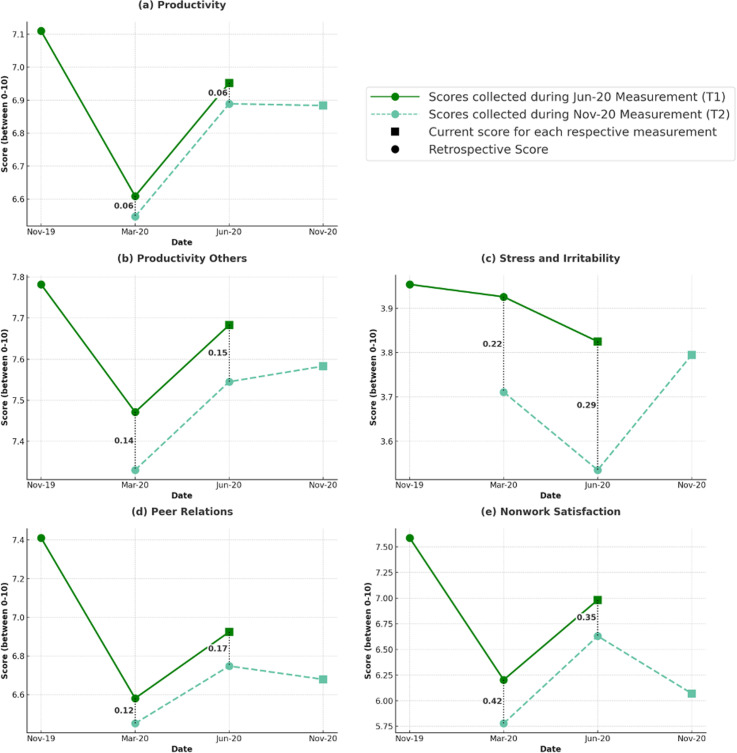
Measurement scores and recollection trends over time per domain. Fig 2 shows the scores collected at June 2020 (T1) and November 2020 (T2), where the square reflects the average score of each respective ‘current’ state, and the dotted icon is the recollected score for each respective measurement. Each domain further includes the delta between the scores of the same period collected during June 2020 and November 2020. Note that means are plotted instead of medians as they better capture small shifts in clustered datasets. Standard deviations (SD) are omitted to avoid between-subject comparisons or misinterpretations of statistical significance, as the analysis focuses on within-sample changes. Standard deviations can be found in Table 1.

The sample average scores of all WFH-HWQ factors for all collected time periods are shown in Table 1. Fig. 2 plots between-sample means as a visualization aid to illustrate general trends, while statistical analyses focus on non-parametric within-sample comparisons. Standard deviations are omitted to avoid suggesting significance in the between-sample trends. Means are plotted instead of medians, as they better capture small shifts in clustered scores. The average trends show that almost all factors dropped immediately after the start of the pandemic in March, and, although improving over time, do not completely recover at the latest measurement in November.

**Table 1:** Working from Home WFH-HWQ scores for all time periods.

		November 2019		March 2020						June 2020						November 2020					
		(1)		(2)		(3)		(4)		(5)		(6)		(7)		(8)		(9)		(10)	
Scored Period		*NT1*		*MT1*		*MT2*		*MT1 = MT2*		*T1*		*RT1*		**T1** = RT1		*T2*		*T2* *** = *** *T1*		*T2 = RT1*	
Collection Period		*T1*		*T1*		*T2*		*–*		*T1*		*T2*		–		*T2*		*–*		*–*	
Variable	*N*	Mdn	*IQR*	Mdn	*IQR*	Mdn	*IQR*	z	p	Mdn	*IQR*	Mdn	*IQR*	z	p	Mdn	*IQR*	z	p	z	p
Productivity	772	7.18	1.30	6.60	1.68	6.70	1.90	1.45	.15	7.05	1.40	6.90	1.45	1.43	.15	6.95	1.65	*0.86*	*.40*	*-0.40*	*.69*
Productivity by Others	742	8.00	1.50	7.50	1.75	7.50	2.00	2.82	.00 *	7.75	1.50	7.75	1.75	3.41	.00**	7.75	1.50	*2.00*	*.05*	*-1.88*	*.06*
Stress and Irritability	772	3.79	2.29	3.86	2.07	3.57	2.29	4.90	.00***	3.71	2.00	3.43	2.07	6.80	.00***	3.64	2.43	*1.38*	*.17*	*-8.13*	.00***
Peer Relations	748	7.67	1.33	6.67	2.00	6.67	2.33	2.01	.04	7.00	1.33	7.00	1.67	3.91	.00***	6.67	1.67	*4.88*	.00***	3.09	.00**
Nonwork Satisfaction	772	7.67	1.33	6.33	2.00	6.00	2.33	6.76	.00***	7.00	1.33	6.67	1.33	6.58	.00***	6.33	2.00	*15.84*	.00***	13.20	.00***

Z scores for pairwise Wilcoxon Signed Rank. Test Significance is corrected by a Bonferroni multiple testing correction: * (.05).01, **(.01).002, and ***(.001).

To test Hypothesis 1 (H1a and H1b), which examines whether participants report significant changes in WFH-related factors across multiple time points within each measurement period, a within-subject Friedman test was conducted. The results confirm significant variation over time in all WFH-related factors for both measurement periods, supporting these hypotheses. For the first measurement, results indicated a significant difference across time points (T1 = MT1 = NT1), *Q*(2) =  202.20, p < .001, with a medium effect size (*W* = .13). For this first data collection in June 2020, post-hoc pairwise comparisons shows that median productivity scores significantly decreased from 7.18 (IQR =  1.30) in November 2019 (NT1) to 6.60 (IQR =  1.68) in March 2020 (MT1) and then slightly increased to 6.70 (IQR =  1.90) in June 2020 (T1). The second measurement at T2 (November 2020) also showed a significant effect of time (MT1 = RT1 = T2; *Q*(2) =  62.64, *p* < .001, W = 0.4). Median productivity scores slightly recovered from 7.05 (IQR =  1.40) in March 2020 (MT2) to 6.90 (IQR =  1.45) in June 2020 (RT1) and 6.95 (IQR =  1.65) in November 2020 (T2). A post-hoc test shows that there is no significant difference between the recollected score of June 2020 (RT1) and November 2020 (T2; *p* = .88). Specifically, T1 = MT1 = NT1 (H1a; *p < *.0004) and T2 = RT1 = MT2 (H1b; *p < *.0004) within each participant are violated for all factors with effect sizes ranging from small (.02) to large (.50; see S2 Table for all within-subject Friedman Tests and corresponding effect sizes for both measurements).

Generally, post-hoc pairwise comparisons indicated that almost all scores differ significantly from each other for all factors in the same measurement (see S3 Fig for Friedman’s Test Post Hoc and Difference Boxplot for a breakdown per factor). Hence, workers in our sample report significant changes over the targeted time period for all WFH-HWQ factors (during both waves). Some within-factor trends and heterogeneity between factors are noteworthy. For instance, productivity and peer relations display a comparable trend: starting high in November 2019, followed by a mild dip in March 2020, and recovering in between those scores in June and November 2020 (*p* < 0.00, W = .13; *p* < 0.00, W = .40, respectively). Interestingly, Stress and Irritability (W = .02) remains relatively stable throughout the start of the pandemic (*p* = .08) but starts to increase from March into June (*p* < .008). Nonwork Satisfaction shows the most movement over time (*p* < .0004; W = .50), mostly decreasing into the pandemic.

Hypothesis 2 tests whether participants’ current state scores differ between the two measurement periods (T1: June 2020 and T2: November 2020) using a within-subject Wilcoxon signed rank test. Results indicate that participants’ current sentiment remains partially unchanged between these two time points. Column 9 of Table 1 shows that only Nonwork Satisfaction and Peer Relations decreased significantly within subjects from T1 to T2 (Wilcoxon Signed Rank Test *z-score* of 4.880, *p < *.0004; and *z* = 15.843, *p < *.0004, respectively). This might indicate a working-from-home leveling-out effect, combined with COVID-19 fatigue: although Productivity and Stress are unchanged, both their personal life quality and Peer Relations are starting to suffer.

### Recollection inaccuracy

Whereas Hypotheses 1 and 2 establish meaningful within-sample variation over time in WFH-related factors, Hypothesis 3a investigates whether participants accurately recollect past experiences. Comparing sample average trends across both waves for the same targeted time periods in Fig. 2 suggests consistent retrospective underestimation. Ideally, accurate recollection would yield similar, nonsignificant differences between T1 and RT1 scores. However, all WFH-HWQ factors appear lower in recollection than in the actual recorded scores, with significant underestimation across almost all factors.

Column 7 of Table 1 supports this observation: all factors except Productivity show statistically significant underestimation in the June recollection (RT1) when recalled in November (for example, Productivity by Others: z =  3.41, p < .002, and for the other factors, p < .0004). Overall, the T1 scores recollected during T2 do not accurately reflect participants’ actual state at T1, revealing a notable bias in retrospective accuracy.

This recollection inaccuracy becomes especially problematic when considered in the context of a hypothetical WFH evaluation based on a single measurement. Instead of comparing the true T1 values with the true T2 values (Column 9), evaluations would then rely solely on Column 10 (the comparison between the recollected T1 values at T and the true T2 values). Comparing these two columns demonstrates that, while participants can somewhat accurately capture the trend for factors like Nonwork Satisfaction and Peer Relations—only slightly underestimating the actual changes—there are noteworthy inconsistencies for other factors. For example, Stress and Irritability is recollected as having shown a big improvement over time (column 10), yet comparison based on actual data (column 9) indicates no significant difference and even hints at a reversed trend. A similar reversion of effect is observed for Productivity by Others, where recollection suggests improvement, but the actual change suggests worsening over time. Note that these latter effects become marginally insignificant after a stringent Bonferroni correction.

These discrepancies illustrate the risk of relying solely on recollected WFH-HWQ scores for evaluation. In some cases, conclusions based on recollection may be the opposite of those based on actual scores, underscoring the need for caution when interpreting retrospective data.

### Inaccuracy mechanism

Hypothesis H3b aimed to explain the source of the observed recollection inaccuracy. H3b examined the relationship between recollected scores (RT1), the target score (T1), and the current score at the time of recollection (T2) to determine whether recollection inaccuracies are systematically biased toward the current state scores. The fact that both the recollected scores and the current scores at T2 are lower hints at an anchoring effect: the current score might influence the recollection more than the score it is supposed to recollect.

The difference between the current and recollection scores ($\left|\Delta \right|$ RT1-T1) is compared to the difference between the target and recollection scores ($\left|\Delta \right|$ RT1-T2) in order to further investigate this inaccuracy implied by the correlation. If the former is consistently smaller than the latter, the recollected score tends to be more biased towards the current score (biased) than to the target score. Table 2 panel A shows the proportion of observations in which the RT1 is closer to T1 (Indicator =  0) or RT1 is closer to T2 (Indicator =  1). Conditional on T1≠T2 and testing deviation from a 50% baseline proportion in the population, binomial tests show that for all WFH-HWQ factors, RT1 is closer to T2 than to T1 (for all, *p* < .0004), further implicating an anchoring bias (see S3 Table for all binomial proportion tests).

**Table 2:** Within-sample Binomial Proportion Accuracy Test (a) and Trend Consistency Comparison (b).

		N	Metric	Average Score	Exact
**Productivity**					
a.	$\left|\Delta \right|$ RT1-T1> $\left|\Delta \right|$ RT1-T2: (Proportion more biased)	737	prop	.62	.00***
b.	*Δ* RT1-T1 = *Δ* MT2-MT1: (Test difference between both recollective Δ's)	772	*Δ*	.00	.83
**Nonwork Satisfaction**					
a.	$\left|\Delta \right|$ RT1-T1> $\left|\Delta \right|$ RT1-T2: (Proportion more biased)	678	prop	.60	.00***
b.	*Δ* RT1-T1 = *Δ* MT2-MT1: (Test difference between both recollective Δ's)	772	*Δ*	.07	.15
**Stress and Irritability**					
a.	$\left|\Delta \right|$ RT1-T1> $\left|\Delta \right|$ RT1-T2: (Proportion more biased)	742	prop	.69	.00***
b.	*Δ* RT1-T1 = *Δ* MT2-MT1: (Test difference between both recollective Δ's)	772	*Δ*	.07	.04
**Peer Relations**					
a.	$\left|\Delta \right|$ RT1-T1> $\left|\Delta \right|$ RT1-T2: (Proportion more biased)	641	prop	.74	.00***
b.	*Δ* RT1-T1 = *Δ* MT2-MT1: (Test difference between both recollective Δ's)	742	*Δ*	.05	.05
**Productivity by Others**					
a.	$\left|\Delta \right|$ RT1-T1> $\left|\Delta \right|$ RT1-T2: (Proportion more biased)	584	prop	.81	.00***
b.	*Δ* RT1-T1 = *Δ* MT2-MT1: (Test difference between both recollective Δ's)	772	*Δ*	.01	.47

For a: Binomial tests exclude the observations in which T1 =  T2. Exact scores indicate the binomial analysis testing the proportion of cases in which RT1 (recollection) is closer (smaller delta) to R2 than to R1 is significantly larger than 50%. For b: the scores indicate the difference between the delta of the June measurement and the delta of the March measurement. Z scores for pairwise Wilcoxon signed rank test significance indicates that the difference in memory inaccuracy between June and March would be different. Significance is corrected by a Bonferroni multiple testing correction: * (.05).01, **(.01).002, and ***(.001).0004

The correlations of the WFH-HWQ factors between the retrospective score, the target score, and the current score are additionally explored. The retrospective scores (RT1) correlate with the target scores (T1;.46 < *r > *.63). This is surprisingly close to the correlations between the within-subject current scores (T1 and T2;.42 < *r* > .59). The correlations between the retrospective score (RT1) and the current in June score (T2) should be lower than the RT1 and T1, yet the correlations are actually the highest (.70 < *r > *.84; see S4 Table for all correlations). The fact that only the RT1-T2 correlations can be considered strong [48] is additional support that recollection bias is caused by the current state scores.

As a final step to examine Hypothesis 3b, a linear regression model estimates the predictive strength both T1 and T2 scores have on RT1 scores whilst allowing to control for individual characteristics. Table 3 shows that the recollected scores are significantly related to both the scores at the time of recollection (T2), as well as the targeted scores T1 for each factor. However, the T2 scores’ coefficients are noticeably and unanimously stronger than the T1 scores’ coefficients (difference in coefficients $(\alpha_{2}-\alpha_{1})$ ranging from.164 to.606 for Productivity and Productivity by Others, respectively). Again, the current score has a greater magnitude impact on the recollection scores than the targeted score.

**Table 3:** Regressions predicting recollected measurement scores.

	(1)	(2)	(3)	(4)	(5)
	RT1 Productivity	RT1 Nonwork Satisfaction	RT1 Stress and Irritability	RT1 Peer Relations	RT1 Productivity by Others
Current Score at T1	0.32 (0.03) ***	0.16 (0.04) ***	0.16 (0.03) ***	0.21 (0.03) ***	0.12 (0.02) ***
Current Score at T2	0.49 (0.03) ***	0.55(0.03) ***	0.67(0.02) ***	0.65 (0.03) ***	0.73(0.02) ***
Household Controls	Yes	Yes	Yes	Yes	Yes
Individual Controls	Yes	Yes	Yes	Yes	Yes
Job Controls	Yes	Yes	Yes	Yes	Yes
Constant	1.10**	1.48**	-0.19	1.11 *	1.52***
	(0.35)	(0.48)	(0.38)	(0.43)	(0.31)
Observations	772	772	772	741	727
R2	0.603	0.528	0.708	0.666	0.734
F Statistic	60.90*** (df:19; 752)	44.28*** (df:19; 752)	95.86*** (df:19; 752)	75.60*** (df:19; 721)	102.43*** (df:19; 707)

Standard errors in parentheses. ^* ^*p* <  0.05, ^**^p <  0.01, ^***^p <  0.001. See S5 Table for the full regression results.

### Structural recollection inconsistency over time

Careful consideration of Fig 2 suggests that, although participants’ recollections do not capture absolute levels accurately, they do retain a reliable sense of the overall trend and historical changes. Specifically, the underestimation pattern identified for June persists into the March recollection (see column 4 of Table 1 for the signed-rank test). This implies that the recollection scores of WFH-HWQ factors follow a systematic bias: participants may consistently remember the relative trend and change over time even when the absolute levels might be biased. For example, a participant who rated their productivity at 8 out of 10 in June may later recall it as 6 out of 10, influenced by their current productivity score of 6.5 out of 10. While this demonstrates a bias in absolute levels, the participant might still correctly remember that their productivity decreased by one point between March and June. Hypothesis 3c investigates whether such relative trends remain consistent across different measurements, indicating that, despite biases in absolute levels, recollections of changes over time may still hold informational value.

First, the within-subject consistency in the discrepancy between two scores of the same period is assessed. Table 2 panel b shows the difference between the average delta of the two scores in June (*Δ* RT1-T1) and the average delta of the two scores in March (*Δ* MT2-MT1) of all factors. None of these differences are significantly different, indicating that, although the absolute levels are structurally different, the difference remains stable over time (see S6 Table for a complete assessment of the retrospective trend consistency comparison).

The OLS models in Table 4 further confirm that the difference between T1 and RT1 is related to the difference between MT1 and MT2 for all WFH-HWQ factors. Specifically, a larger difference between RT1 and T1 scores predicts a larger difference between MT1 and MT2 ($\beta_{1}$ ranging from 0.22 to 0.61, *p* < .001). Together, these findings indicate that recollection inaccuracies are not random but structurally influence recollection similarly over time. Although recollections may be biased toward the current state scores, participants consistently recall relative changes between periods, implying that trend information remains reliable even if absolute levels are biased.

**Table 4:** Regressions of predictive trend in measurement scores over time.

	(1)	(2)	(3)	(4)	(5)
	$\left|\Delta \right|$ Productivity (MT1 – MT2)	$\left|\Delta \right|$ Nonwork Satisfaction (MT1 – MT2)	$\left|\Delta \right|$ Stress and Irritability (MT1 – MT2)	$\left|\Delta \right|$ Peer Relations (MT1 – MT2)	$\left|\Delta \right|$ Productivity by others (MT1 – MT2)
$\left|\Delta \right|$ RT1– T1	0.22(0.03)***	0.39(0.03)***	0.53(0.03)***	0.53(0.03)***	0.61(0.03)***
Household Controls	Yes	Yes	Yes	Yes	Yes
Individual Controls	Yes	Yes	Yes	Yes	Yes
Job Controls	Yes	Yes	Yes	Yes	Yes
Constant	0.54	0.78	0.20	0.86 *	0.91 *
	(0.32)	(0.47)	(0.35)	(0.43)	(0.37)
Observations	772	772	772	741	727
R2	0.079	0.174	0.311	0.281	0.341
F Statistic	3.58*** (df:18; 753)	8.80*** (df:18; 753)	18.95*** (df:18; 753)	15.65*** (df:18; 722)	20.31*** (df:18; 708)

Standard errors in parentheses. ^* ^*p* <  0.05, ^**^p <  0.01, ^***^p <  0.001. See S7 Table for the full regression results.

## Conclusion

The prevalent use of self-reported productivity and satisfaction as key metrics for gauging working-from-home success has influenced the formulation of many detailed WFH policies, yet accumulating evidence indicates a persistent divergence between perceived and quantifiable WFH productivity, with the latter frequently exhibiting a downward trend [13–15]. This paper shows evidence that self-reported working-from-home evaluations are subject to inaccurate recollection: the recollection of past scores is structurally being anchored on current scores. This insight remains relevant in the ongoing discussion, where the high-profile evaluation of WFH and the future of work continues to heavily rely on one’s own or others’ perceived performance [16].

First, homeworkers in this sample report significant changes over time in all self-reported productivity-related factors during the shift to home during the 2020 pandemic period. Memory research suggests that the accuracy of autobiographical recollection is often compromised by systematic biases, particularly in periods of disruption or instability [21,25–28]. By exploring the consistency of these self-reports using repeated-measures approach, the working-from-home evaluations are shown to be subject to an anchoring bias where the recollection scores are more similar to the current state scores than to the targeted past state scores. This aligns with prior research on anchoring in memory [42,43], which suggests that individuals disproportionately use present states as reference points when recalling past states. In some cases, comparisons based solely on recollected data can yield conclusions about changes over time that are opposite to those based on actual changes over time, underscoring the need for caution when interpreting retrospective self-reports.

Retrospective trends further imply that the comparative recollection is structural: the current state primarily biases the absolute reference point without gravely affecting the recalled changes over time amidst a volatile, pandemic-ridden period. Specifically, the trends for both measurements remain consistent, even when the current state scores serve as an anchoring reference point biasing the absolute score of all recollection scores. This suggests that memory is not fundamentally impaired, and the trend remains accurate and informative, even when the absolute levels suffer from an anchoring bias. These results support the idea that memory biases can be both systematic and predictable [20,49]. Nevertheless, the absolute retrospective scores are influenced by the current state scores by an a priori unknown degree.

Together, this paper is the first to address and provide a scientific explanation within a robust theoretical framework for the highly debated and recurring discrepancy between optimistic self-reported WFH evaluations and increasingly pessimistic objective measurements. Given that research on recollection biases suggests that perceived changes can be exaggerated or minimized depending on the context and perceived goals [37–39], these findings provide a new lens for interpreting WFH self-assessments. The sole reliance on self-reported WFH productivity evaluations does not seem justified without acknowledging human recollection inaccuracy and could partially explain the observed discrepancy between objective and subjective WFH evaluations.

## Limitations

This study, while offering valuable insights into the effects of work-from-home (WFH) on productivity and stress, has several limitations that warrant consideration. First, the participant pool comprises solely Dutch workers capable of remote work and may not represent the broader global workforce. This limitation is crucial, as cultural, economic, and workplace differences could significantly influence the outcomes related to work-from-home (WFH) practices. Similarly, the context of the COVID-19 pandemic presents both time- and demographic-specific limitations on generalizability. The unique circumstances of the pandemic, including heightened stress levels and unprecedented changes in work practices, might have influenced the perception of work or recollection accuracy. Consequently, further investigation should confirm that these findings are not limited to the specific demographic, the onset of the pandemic period, and the geographical context.

Second, the timing of the two measurement periods, while carefully selected to ensure comparability in work-from-home policies and social distancing restrictions, poses limitations. The first wave occurred during a period of easing restrictions, while the second coincided with the reintroduction of stricter policies. Although the timing minimized the influence of external factors and capitalized on the (normally not volatile) changes in self-reported productivity independent of job-specific changes, subtle differences in time-specific pandemic context could have influenced participants’ recollections. Another limitation of attempting to select comparable data collection periods is the inability to investigate how the length of the reporting period (i.e., the time elapsed between the event and recollection) influences accuracy. Longer reporting periods might reinforce biases due to memory decay, while shorter periods could reduce the scope for meaningful recall of changes. Future research could explore the role timeframes play for accurate recollection and whether WFH memory reliability is poor under all conditions.

Third, the lack of objective productivity metrics alongside self-reported data limits the study's ability to validate or cross-reference the reported perceptions of productivity. Although this study focuses on recollection accuracy, it fundamentally examines differences between two measures of self-report, rather than a direct comparison with objective productivity metrics. While the relevance of the questionnaire is emphasized by the limited availability of objective productivity data, future research should explore the accuracy gap between realized and self-reported scores in the work domain. Furthermore, reliance on self-reported data introduces potential biases, such as social desirability, which may affect the accuracy and reliability of responses, particularly regarding productivity and stress levels.

Fourth, this study cannot distinguish between technical and mental anchoring in the observed recollection bias. A survey design fully dependent on self-reports cannot disentangle whether prior responses (technical anchoring) or specific emotional or event-based cues (mental anchoring) primarily influence recollection inaccuracy. While qualitative interviews or objective measures could reduce the former, newly uncovered discrepancies between objective and self-reported productivity in WFH suggest the latter is significant. A deeper understanding of these dynamics is critical for understanding how productivity and stress levels evolve over time.

Finally, the study's statistical approach, being largely correlational, limits causal interpretation. In spite of a multitude of confirmatory approaches and attempts to control for omitted variables, the absence of experimental manipulation means that the findings indicate associations and are limited in the ability to definitively identify causal relationships. Although reverse causality is unlikely based on memory literature, future research should employ experimental or quasi-experimental designs to strengthen causal interpretations of the relationship between WFH, productivity, and stress recollection over time.

### Implications

First, these findings show that the sole reliance on self-reported WFH productivity evaluations does not seem justified without acknowledging human recollection inaccuracy. This is paramount for the evaluation and subsequent continuation of work-from-home and has far-reaching consequences for society at large. The mostly self-reported success of WFH has already instigated change: (prospective) employees demand WFH opportunities [50], firms rapidly introduce invasive office policies [51], and resulting residential relocation preferences are even reshaping urban environments [52,53]. Although this paper is agnostic about the future of WFH and its objective impact on productivity, it is the first to advocate caution when interpreting self-reported recollected work-related scores in the working-from-home domain. Sole reliance on biased accounts without acknowledging and correcting for the self-reported inaccuracy of work-from-home success could fundamentally undermine WFH’s future assessment and render the consequential anticipatory efforts suboptimal or even counterproductive.

Second, this paper contributes to the emerging body of self-reported assessment research in the absence of objective observations. Self-reports have been scrutinized in the psychological context, but the psychological limitations of self-report within the organizational and economic research domain have largely remained overlooked. This paper reconciles the large body of behavioral research with the applied setting of economically relevant productivity. These findings highlight the necessity of caution and critical review when utilizing self-reported studies, as the nature of human behavior has the potential to reflect a non-existing or opposite change even within the productivity domain [39,54].

## Supporting information

S1 Table Descriptives Table.
(DOCX)

S2 Table Extended Mantel-Haenszel Stratified Test of Association – Friedman’s Test.
(DOCX)

S1 Fig Friedman’s Test Post Hoc Coordinates plots and differences box plot.
(TIF)

S3 Table WFH-HWQ Binomial Proportion Accuracy Test.
(DOCX)

S4 Table Correlations between WFH-HWQ factors scores on T1, T2.
(DOCX)

S5 Table Recollection Prediction Regression for all factors.
(DOCX)

S6 Table WFH-HWQ Retrospective Trend Consistency Comparison.
(DOCX)

S7 Table Delta Trends Regression for all factors.
(DOCX)
