# Supplementary material for: Productivity and stress recollection inaccuracy: Anchoring effects in work-from-home evaluation
Source: PLoS One. 2025 Apr 3;20(4):e0320959. doi: 10.1371/journal.pone.0320959 (PMC11967955; doi:10.1371/journal.pone.0320959)
Supplement: S1 Table — (DOCX) [file pone.0320959.s001.docx]

**S1 Table.** Descriptives Table

|  | Mean (SD) / % |
| --- | --- |
| **Individual Characteristics** |  |
| Age (years) | 44.27 (12.46) |
| Female | 42% |
| Income (annual in €) |  |
| Minimum wage (less than 11,000) | 2% |
| below modal (11-23k) | 11% |
| Modals (24-34k) | 19% |
| 1-2x modal (34-56k) | 32% |
| 2x modal or more (56k) | 22% |
| don’t know/ don’t want to say | 15% |
| Education Level |  |
| Low (postsecondary vocational education or lower-level high school) | 7% |
| Mid (post-secondary vocational degree, undergraduate  education, or higher level of high school) | 39% |
| High (undergraduate degree or higher) | 54% |
|  |  |
| **Household Characteristics** |  |
| Children Home during Office Hours |  |
| Always | 7% |
| Sometimes | 32% |
| Never | 15% |
| No Children in Household | 47% |
| Household size (including respondent) | 2.65 (1.26) |
|  |  |
| **Job-related Characteristics** |  |
| Work suitable to perform from home  (scale 1-10; higher is more suitable) | 7.46 (2.19) |
| Prior to the lockdown, did you have experience with work-from-home (% yes) | 53% |
| Does your job generally use deadlines?  (scale 1-5; higher is more deadline usage) | 3.50 (1.13) |
| Do you experience control from your direct supervisor  (scale 1-5; higher is more experienced control) | 3.02 (1.33) |
