# Supplementary material for: Productivity and stress recollection inaccuracy: Anchoring effects in work-from-home evaluation
Source: PLoS One. 2025 Apr 3;20(4):e0320959. doi: 10.1371/journal.pone.0320959 (PMC11967955; doi:10.1371/journal.pone.0320959)
Supplement: S2 Table — (DOCX) [file pone.0320959.s002.docx]

**S2 Table.** Extended Mantel-Haenszel Stratified Test of Association – Friedman’s Test

|  | June 2020 measurement | | | | | | | |  | November 2020 measurement | | | | | | | |  |
| --- | --- | --- | --- | --- | --- | --- | --- | --- | --- | --- | --- | --- | --- | --- | --- | --- | --- | --- |
|  | (1) | | (2) | | (3) | | (4) | |  | (5) | | (6) | | (7) | | (8) | |  |
|  | *NT1* | | *MT1* | | ***T1*** | |  | |  | *MT2* | | *RT1* | | ***T2*** | |  | |  |
| Variable | Mdn | *IQR* | Mdn | *IQR* | Mdn | *IQR* | Q | *p* | *W* | Mdn | *IQR* | Mdn | *IQR* | Mdn | *IQR* | Q | *p* | *W* |
| Productivity | 7.18 | 1.30 | 6.60 | 1.68 | 6.70 | 1.90 | 202.20 (df:2) | .00*** | .13 | 7.05 | 1.40 | 6.90 | 1.45 | 6.95 | 1.65 | 62.64 (df:2) | .00*** | .04 |
| Productivity by others | 8.00 | 1.50 | 7.50 | 1.75 | 7.50 | 2.00 | 159.22 (df:2) | .00*** | .11 | 7.75 | 1.50 | 7.75 | 1.75 | 7.75 | 1.50 | 50.71 (df:2) | .00*** | .03 |
| Stress and Irritability | 3.79 | 2.29 | 3.86 | 2.07 | 3.57 | 2.29 | 26.76 (df:2) | .00*** | .02 | 3.71 | 2.00 | 3.43 | 2.07 | 3.64 | 2.43 | 86.74 (df:2) | .00*** | .06 |
| Peer Relations | 7.67 | 1.33 | 6.67 | 2.00 | 6.67 | 2.33 | 591.71 (df:2) | .00*** | .40 | 7.00 | 1.33 | 7.00 | 1.67 | 6.67 | 1.67 | 66.97 (df:2) | .00*** | .05 |
| Nonwork Satisfaction | 7.67 | 1.33 | 6.33 | 2.00 | 6.00 | 2.33 | 773.05 (df:2) | .00*** | .50 | 7.00 | 1.33 | 6.67 | 1.33 | 6.33 | 2.00 | 370.67 (df:2) | .00*** | .24 |

**Note.** Q scores for non-parametric repeated-measure pairwise ANOVA alternative. Effect size in Kendall’s W: small is W <0.1, Medium 0.1 < W < 0.3, and Large W>0.3 Friedman Test. Significance is corrected by a Bonferroni multiple testing correction: *(.05).01, **(.01).002, and ***(.001).0004.
