# Supplementary figures and images for: Productivity and stress recollection inaccuracy: Anchoring effects in work-from-home evaluation
Source: PLoS One. 2025 Apr 3;20(4):e0320959. doi: 10.1371/journal.pone.0320959 (PMC11967955; doi:10.1371/journal.pone.0320959)

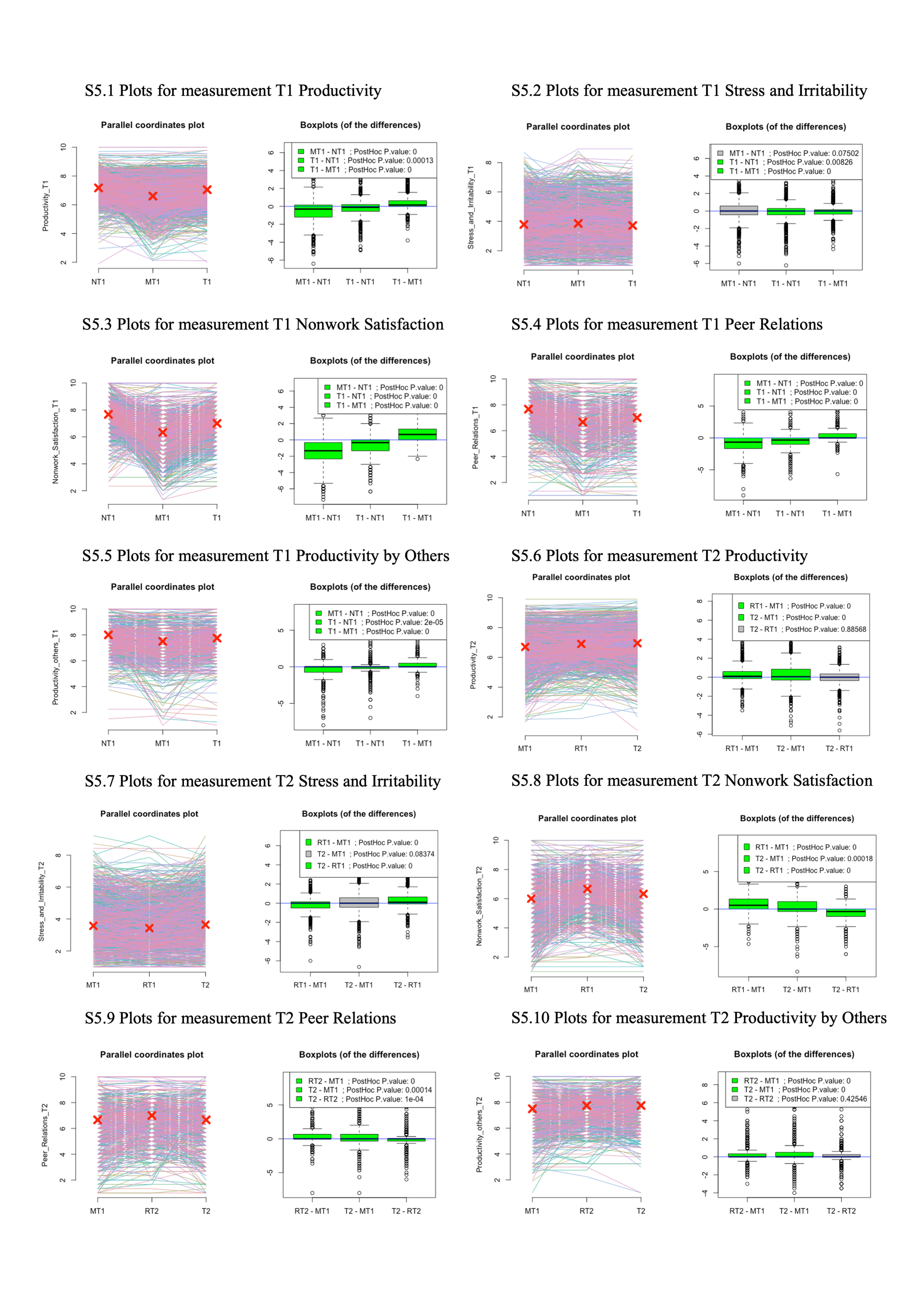

Supplement: S1 Fig — (TIF) [file pone.0320959.s003.tif]
