# Supplementary material for: Productivity and stress recollection inaccuracy: Anchoring effects in work-from-home evaluation
Source: PLoS One. 2025 Apr 3;20(4):e0320959. doi: 10.1371/journal.pone.0320959 (PMC11967955; doi:10.1371/journal.pone.0320959)
Supplement: S3 Table — (DOCX) [file pone.0320959.s004.docx]

**S4 Table.** WFH-HWQ Binomial Proportion Accuracy test

|  |  | Observed prop | Test prop | Exact (two-sided) |
| --- | --- | --- | --- | --- |
| Productivity | $\left\vert\Delta\right\vert$RT1-T1 < $\left\vert\Delta\right\vert$ RT1-T2: (more accurate) | .38 | .50 | .000*** |
| N=737 | $\left\vert\Delta\right\vert$RT1-T1 > $\left\vert\Delta\right\vert$ RT1-T2: (more biased) | .62 |  |  |
|  |  |  |  |  |
| Nonwork Satisfaction | $\left\vert\Delta\right\vert$RT1-T1 < $\left\vert\Delta\right\vert$ RT1-T2: (more accurate) | .40 | .50 | .000*** |
| N=678 | $\left\vert\Delta\right\vert$RT1-T1 > $\left\vert\Delta\right\vert$ RT1-T2: (more biased) | .60 |  |  |
|  |  |  |  |  |
| Stress and Irritability | $\left\vert\Delta\right\vert$RT1-T1 < $\left\vert\Delta\right\vert$ RT1-T2: (more accurate) | .31 | .50 | .000*** |
| N=742 | $\left\vert\Delta\right\vert$RT1-T1 > $\left\vert\Delta\right\vert$ RT1-T2: (more biased) | .69 |  |  |
|  |  |  |  |  |
| Peer Relations | $\left\vert\Delta\right\vert$RT1-T1 < $\left\vert\Delta\right\vert$ RT1-T2: (more accurate) | .26 | .50 | .000*** |
| N=641 | $\left\vert\Delta\right\vert$RT1-T1 > $\left\vert\Delta\right\vert$ RT1-T2: (more biased) | .74 |  |  |
|  |  |  |  |  |
| Productivity by Others | $\left\vert\Delta\right\vert$RT1-T1 < $\left\vert\Delta\right\vert$ RT1-T2: (more accurate) | .19 | .50 | .000*** |
| N=584 | $\left\vert\Delta\right\vert$RT1-T1 > $\left\vert\Delta\right\vert$ RT1-T2: (more biased) | .81 |  |  |

Binomial tests exclude the observations in which T1 = T2. Exact scores indicate the binomial analysis and are corrected by

a Bonferroni multiple testing correction: *(.05).01, **(.01).002, and ***(.001).0004
