# Supplementary material for: Productivity and stress recollection inaccuracy: Anchoring effects in work-from-home evaluation
Source: PLoS One. 2025 Apr 3;20(4):e0320959. doi: 10.1371/journal.pone.0320959 (PMC11967955; doi:10.1371/journal.pone.0320959)
Supplement: S5 Table — (DOCX) [file pone.0320959.s006.docx]

**S5 Table.** Correlations between WFH-HWQ factors scores on T1, T2, and RT1

| Variables | (1) | (2) | (3) | (4) | (5) | (6) | (7) | (8) | (9) | (10) | (11) | (12) | (13) | (14) | (15) |
| --- | --- | --- | --- | --- | --- | --- | --- | --- | --- | --- | --- | --- | --- | --- | --- |
| (1) Productivity T2 | 1 |  |  |  |  |  |  |  |  |  |  |  |  |  |  |
| (2) Productivity T1 | 0.59 | 1 |  |  |  |  |  |  |  |  |  |  |  |  |  |
| (3) Productivity RT1 | 0.72 | 0.63 | 1 |  |  |  |  |  |  |  |  |  |  |  |  |
| (4) Productivity by others T2 | 0.61 | 0.41 | 0.50 | 1 |  |  |  |  |  |  |  |  |  |  |  |
| (5) Productivity by others T1 | 0.34 | 0.56 | 0.37 | 0.42 | 1 |  |  |  |  |  |  |  |  |  |  |
| (6) Productivity by others RT1 | 0.45 | 0.41 | 0.59 | 0.84 | 0.45 | 1 |  |  |  |  |  |  |  |  |  |
| (7) Stress and irritability T2 | -0.54 | -0.40 | -0.40 | -0.26 | -0.19 | -0.16 | 1 |  |  |  |  |  |  |  |  |
| (8) Stress and irritability T1 | -0.32 | -0.48 | -0.31 | -0.19 | -0.23 | -0.17 | 0.59 | 1 |  |  |  |  |  |  |  |
| (9) Stress and irritability RT1 | -0.38 | -0.39 | -0.46 | -0.22 | -0.23 | -0.22 | 0.83 | 0.59 | 1 |  |  |  |  |  |  |
| (10) Peer Relations T2 | 0.49 | 0.39 | 0.40 | 0.39 | 0.32 | 0.31 | -0.34 | -0.21 | -0.28 | 1 |  |  |  |  |  |
| (11) Peer Relations T1 | 0.30 | 0.47 | 0.31 | 0.28 | 0.41 | 0.28 | -0.23 | -0.28 | -0.25 | 0.57 | 1 |  |  |  |  |
| (12) Peer Relations RT1 | 0.33 | 0.38 | 0.49 | 0.31 | 0.34 | 0.39 | -0.23 | -0.20 | -0.28 | 0.79 | 0.59 | 1 |  |  |  |
| (13) Nonwork Relations T2 | 0.36 | 0.27 | 0.29 | 0.24 | 0.08 | 0.19 | -0.25 | -0.12 | -0.19 | 0.48 | 0.28 | 0.39 | 1 |  |  |
| (14) Nonwork Relations T1 | 0.30 | 0.40 | 0.30 | 0.25 | 0.19 | 0.22 | -0.25 | -0.26 | -0.25 | 0.37 | 0.49 | 0.38 | 0.50 | 1 |  |
| (15) Nonwork Relations RT1 | 0.23 | 0.23 | 0.37 | 0.19 | 0.10 | 0.21 | -0.16 | -0.10 | -0.24 | 0.37 | 0.25 | 0.43 | 0.70 | 0.46 | 1 |

Table shows the correlations between all WFH-HWQ factor November (T2), June (T1), and retrospective June (RT1) scores. Ideally, T1 would correlate with T2 with stable

sentiment, and regardless of sentiment change, RT1 would correlate higher with T1 (target score) than with T2 (the current score). Therefore, the Table coloring indicates the

consistency correlation (T1 and T2) in green, the targeted retrospective correlation grey (RT1 and T1), and the carryover correlation (RT1 and T2) in red
