# Supplementary material for: Productivity and stress recollection inaccuracy: Anchoring effects in work-from-home evaluation
Source: PLoS One. 2025 Apr 3;20(4):e0320959. doi: 10.1371/journal.pone.0320959 (PMC11967955; doi:10.1371/journal.pone.0320959)
Supplement: S6 Table — (DOCX) [file pone.0320959.s007.docx]

**S6 Table.** Recollection Prediction Regression for all factors

| Dependent variable: Recollected RT1 HWQ factor score | | | | | |
| --- | --- | --- | --- | --- | --- |
|  | RT1  Productivity | RT1  Nonwork Satisfaction | RT1  Stress and Irritability | RT1  Peer Relations | RT1  Productivity by Others |
| Current Score at T1 | 0.32 (0.03) *** | 0.16 (0.04) *** | 0.16 (0.03) *** | 0.21 (0.03) *** | 0.12 (0.02) *** |
| Current Score at T2 | 0.49 (0.03) *** | 0.55 (0.03) *** | 0.67 (0.02) *** | 0.65 (0.03) *** | 0.73 (0.02) *** |
|  |  |  |  |  |  |
|  |  |  |  |  |  |
| **Individual Characteristics** |  |  |  |  |  |
| Age (years) | 0.01(0.00) | 0.00(0.00) | 0.00(0.00) | 0.00(0.00) | -0.00(0.00) |
| Female | -0.06(0.06) | 0.05(0.08) | -0.02(0.06) | -0.06(0.07) | -0.06(0.05) |
| Income (Baseline: Minimum Wage) |  |  |  |  |  |
| below modal (11-23k) | -0.33(0.19) | -0.20(0.26) | 0.70(0.21)** | -0.03(0.24) | -0.30(0.17) |
| modal (24-34k) | -0.20(0.19) | 0.12(0.25) | 0.64(0.21)** | -0.10(0.23) | -0.35(0.16)* |
| 1-2x modal (34-56k) | -0.18(0.18) | 0.08(0.24) | 0.51(0.20)* | -0.10(0.22) | -0.28(0.16) |
| 2x modal or more (56k) | -0.24(0.19) | 0.13(0.25) | 0.48(0.21)* | -0.04(0.23) | -0.39(0.16)* |
| don’t know/ don’t want to say | -0.24(0.19) | -0.09(0.25) | 0.69(0.21)** | -0.25(0.23) | -0.40(0.16)* |
| Education Level (Baseline: Low) |  |  |  |  |  |
| Mid (post-secondary vocational degree, undergraduate  education, or higher level of high school) | -0.02(0.11) | 0.05(0.15) | -0.07(0.12) | -0.06(0.14) | -0.24*(0.09) |
| High (undergraduate degree or higher) | 0.02(0.11) | 0.28(0.15) | -0.09(0.13) | -0.02(0.14) | -0.25*(0.10) |
|  |  |  |  |  |  |
| **Household Characteristics** |  |  |  |  |  |
| Children Home during Office Hours (baseline: yes always) |  |  |  |  |  |
| Yes, Sometimes | 0.02(0.11) | -0.01(0.14) | 0.05(0.12) | 0.04(0.13) | 0.08(0.09) |
| No, Never | -0.05(0.13) | 0.05(0.17) | -0.00(0.14) | -0.07(0.15) | -0.03(0.11) |
| No Childern | -0.00(0.12) | 0.05(0.16) | 0.12(0.14) | 0.13(0.15) | 0.00(0.10) |
| Household size (including respondent) | -0.00(0.03) | 0.01(0.04) | 0.04(0.03) | -0.00(0.03) | -0.03(0.02) |
|  |  |  |  |  |  |
| **Job-related Characteristics** |  |  |  |  |  |
| Work suitable to perform from home | 0.03(0.01)* | 0.04(0.02)* | -0.03(0.01)* | 0.00(0.01) | 0.02(0.01) |
| Prior to the lockdown, did you have experience with work-from-home (Baseline: No) | -0.04(0.05) | -0.16(0.07)* | 0.09(0.06) | 0.07(0.07) | 0.01(0.05) |
| Does your job generally use deadlines? | 0.00(0.02) | 0.02(0.03) | -0.02(0.03) | -0.05(0.03) | 0.01(0.02) |
| Do you experience control from your direct supervisor? | 0.01(0.02) | 0.00(0.03) | 0.02(0.02) | -0.02(0.03) | 0.02(0.02) |
|  |  |  |  |  |  |
| Constant | 1.10** | 1.48** | -0.19 | 1.11* | 1.52*** |
|  | (0.35) | (0.48) | (0.38) | (0.43) | (0.31) |
| Observations | 772 | 772 | 772 | 741 | 727 |
| R2 | 0.603 | 0.528 | 0.708 | 0.666 | 0.734 |
| F Statistic | 60.90*** (df:19; 752) | 44.28*** (df:19; 752) | 95.86*** (df:19; 752) | 75.60*** (df:19; 721) | 102.43*** (df:19; 707) |
| *Note.* **p*<0.1, ***p*<0.05, ****p*<0.01. Standard errors in parentheses. For Education low descriptions, see Appendix Tabel S1 | | | | | |
