# Supplementary material for: Productivity and stress recollection inaccuracy: Anchoring effects in work-from-home evaluation
Source: PLoS One. 2025 Apr 3;20(4):e0320959. doi: 10.1371/journal.pone.0320959 (PMC11967955; doi:10.1371/journal.pone.0320959)
Supplement: S7 Table — (DOCX) [file pone.0320959.s008.docx]

**S7 Table.** WFH-HWQ Retrospective Trend Consistency Comparison

|  |  |  |  |  |  |  |  |
| --- | --- | --- | --- | --- | --- | --- | --- |
|  |  | N | Mean | SD | Min | Max | exact |
|  |  |  |  |  |  |  |  |
| Productivity | $\Delta$RT1-T1 | 772 | -.06 | .93 | -4.15 | 3.60 | .83 |
|  | $\Delta$MT2-MT1 | 772 | -.06 | 1.06 | -4.20 | 4.55 |  |
|  |  |  |  |  |  |  |  |
| Nonwork Satisfaction | $\Delta$RT1-T1 | 772 | -.35 | 1.32 | -6.00 | 3.67 | .15 |
|  | $\Delta$MT2-MT1 | 772 | -.42 | 1.67 | -6.33 | 5.00 |  |
|  |  |  |  |  |  |  |  |
| Stress and Irritability | $\Delta$RT1-T1 | 772 | -.29 | 1.30 | -4.29 | 5.36 | .04 |
|  | $\Delta$MT2-MT1 | 772 | -.22 | 1.37 | -5.43 | 5.57 |  |
|  |  |  |  |  |  |  |  |
| Peer Relations | $\Delta$RT1-T1 | 741 | -.17 | 1.30 | -5.00 | 5.00 | .05 |
|  | $\Delta$MT2-MT1 | 742 | -.12 | 1.51 | -7.00 | 7.67 |  |
|  |  |  |  |  |  |  |  |
| Productivity by Others | $\Delta$RT1-T1 | 727 | -.14 | 1.15 | -3.50 | 7.50 | .47 |
|  | $\Delta$MT2-MT1 | 727 | -.15 | 1.33 | -4.75 | 6.75 |  |
|  |  |  |  |  |  |  |  |

**Note.** Z scores for pairwise Wilcoxon Signed Rank Test Significance is corrected by a Bonferroni multiple testing correction: *(.05).01, **(.01).002, and ***(.001).0004
